# Integrated multi-omic analysis of host-microbiota interactions in acute oak decline

**DOI:** 10.1186/s40168-018-0408-5

**Published:** 2018-01-30

**Authors:** Martin Broberg, James Doonan, Filip Mundt, Sandra Denman, James E. McDonald

**Affiliations:** 1 School of Biological Sciences, Bangor University, Memorial Building, Deiniol Road, Bangor, Gwynedd LL57 2UW UK; 2 Swedish University of Agricultural Sciences, Almas Alle 8, 750 07 Uppsala, Sweden; 3 Broad Institute, 415 Main St., Cambridge, MA 02142 USA; 4 Forest Research, Centre for Forestry and Climate Change, Alice Holt Lodge, Farnham, Surrey GU10 4LH UK

**Keywords:** Holobiont, Multi-omics, Meta-omics, Microbiome, Microbiota, Plant-microbiome interactions

## Abstract

**Background:**

Britain’s native oak species are currently under threat from acute oak decline (AOD), a decline-disease where stem bleeds overlying necrotic lesions in the inner bark and larval galleries of the bark-boring beetle, *Agrilus biguttatus*, represent the primary symptoms. It is known that complex interactions between the plant host and its microbiome, i.e. the holobiont, significantly influence the health status of the plant. In AOD, necrotic lesions are caused by a microbiome shift to a pathobiome consisting predominantly of *Brenneria goodwinii*, *Gibbsiella quercinecans*, *Rahnella victoriana* and potentially other bacteria. However, the specific mechanistic processes of the microbiota causing tissue necrosis, and the host response, have not been established and represent a barrier to understanding and managing this decline.

**Results:**

We profiled the metagenome, metatranscriptome and metaproteome of inner bark tissue from AOD symptomatic and non-symptomatic trees to characterise microbiota-host interactions. Active bacterial virulence factors such as plant cell wall-degrading enzymes, reactive oxygen species defence and flagella in AOD lesions, along with host defence responses including reactive oxygen species, cell wall modification and defence regulators were identified. *B*. *goodwinii* dominated the lesion microbiome, with significant expression of virulence factors such as the phytopathogen effector *avrE*. A smaller proportion of microbiome activity was attributed to *G*. *quercinecans* and *R*. *victoriana*. In addition, we describe for the first time the potential role of two previously uncharacterised Gram-positive bacteria predicted from metagenomic binning and identified as active in the AOD lesion metatranscriptome and metaproteome, implicating them in lesion formation.

**Conclusions:**

This multi-omic study provides novel functional insights into microbiota-host interactions in AOD, a complex arboreal decline disease where polymicrobial-host interactions result in lesion formation on tree stems. We present the first descriptions of holobiont function in oak health and disease, specifically, the relative lesion activity of *B*. *goodwinii*, *G*. *quercinecans*, *Rahnella victoriana* and other bacteria. Thus, the research presented here provides evidence of some of the mechanisms used by members of the lesion microbiome and a template for future multi-omic research into holobiont characterisation, plant polymicrobial diseases and pathogen defence in trees.

**Electronic supplementary material:**

The online version of this article (10.1186/s40168-018-0408-5) contains supplementary material, which is available to authorized users.

## Background

The current global spread of tree diseases and pests are threatening the diversity, visual aesthetics and ecological roles of forests [1]. Oak trees (*Quercus robur* and *Quercus petraea*) constitute an iconic and fundamental part of British forests and are currently under threat from an episode of acute oak decline (AOD), a complex decline-disease resulting from a combination of several biotic and abiotic factors [2]. AOD is potentially lethal to the trees and has been primarily spreading through southern and central England, and southern Wales [3]. The decline-disease shares similarities to oak declines reported in mainland Europe [4]. The primary symptoms of AOD consist of stem bleeds, cracks in the outer bark plates, necrotic tissue in the underlying inner bark and larval galleries of the bark-boring beetle *Agrilus biguttatus* in close proximity to the lesions [5, 6]*.* Recently, we identified several bacterial species, *Brenneria goodwinii*, *Gibbsiella quercinecans* and *Rahnella victoriana*, as key members of the AOD lesion microbiome [2, 4] and demonstrated through infectivity studies that *B*. *goodwinii* and *G*. *quercinecans* cause tissue maceration in the inner bark, the primary symptom of AOD [4]. The *Enterobacteriaceae*, to which *G*. *quercinecans* belongs, *Yersiniaceae* to which *R*. *victoriana* belongs and *Pectobacteriaceae*, to which *B*. *goodwinii* belongs, include widespread and well-characterised plant-associated bacteria, acting either beneficially or as phytopathogens, with common virulence-associated features such as plant cell wall-degrading enzymes (PCWDEs), protein transport systems, effector proteins, motility and toxin production [7]. However, the specific functional mechanisms that underlie and trigger lesion formation by *B*. *goodwinii* and *G*. *quercinecans* are unknown and currently represent a significant barrier to understanding the aetiology of AOD, and ultimately in managing the decline. Furthermore, the roles of *R*. *victoriana* and other abundant members of the AOD lesion microbiome, as symbionts or pathogens, and their interactions with the host or other members of the microbiota are poorly understood. The AOD microbiome therefore represents an excellent model system that provide new insights into plant holobionts. Here, we performed multi-omic analysis of host-microbiota interactions in non-symptomatic and AOD symptomatic oak in order to understand the functional shifts and mechanistic processes underlying polybacterial lesion formation and host defences in AOD. The results will therefore provide insights into the triggers and functional mechanisms of lesion formation, allow identification of key causal agents of the decline, identify candidate markers for rapid field diagnostic tests and ascertain potential markers for future breeding of resistant oak, all of which will contribute to future management of the decline.

The microbiome is increasingly recognised as crucial to the understanding of plant health [8]. Indeed, microbiomes have been characterised across the roots, stems and leaves of model plants, transforming our understanding of beneficial and deleterious interactions within the holobiont [9–11]. While the stem microbiome has been generally less explored than the phylloplane (foliage) or the rhizosphere in trees, it may perform important functions, for example nitrogen fixation [9]. Holistic analysis of the microbiome and its interactions with the host by high-throughput genomics, transcriptomics and proteomics (multi-omics) has become highlighted as an important area in plant research [8, 12, 13]. Metagenomics provides gene inventories of environmental samples that are/can be linked to specific functions, while metatranscriptomics and metaproteomics demonstrate gene activity [14–16]. These methods serve to identify and profile the phenotype of the holobiont, a term increasingly used to describe the combination of host and microbiome, across time and space, with metagenomics identifying the hologenome of the holobiont [17, 18]. Thus, it is important to obtain information and evidence showing the mechanisms of functional change as the healthy microbiome of oak succumbing to AOD shifts into an AOD microbiome, and where otherwise, benign microbes may become opportunistic pathobionts and increase/change in activity [4].

Our study had four aims: (1) to expand upon the known taxonomic composition of AOD-associated microbiota by performing bioinformatic genome reconstruction from metagenomic data of organisms undetected by previous taxonomic analyses, in order to gain further clarity of the composition of the microbiome; (2) to identify the functional activity, host-microbe interactions and relative importance of *B*. *goodwinii*, *G*. *quercinecans*, *R*. *victoriana* and other bacteria in the AOD microbiome in lesion formation using their complete genome sequences for mapping of our meta-omic data; (3) to investigate the possibility of functional genes belonging to other bacteria of interest identified by Denman et al. [4] in AOD lesion formation and host infection dynamics; and (4) to investigate the oak host response in AOD, in order to understand the aetiology and host reaction to infection in active AOD lesions. To achieve these aims, we performed the first parallel multi-omic analysis of inner bark from non-symptomatic oak trees (no symptoms of poor health) and lesion tissue from oaks affected by AOD (clearly visible active stem lesions and reduced crowns), demonstrating that *B*. *goodwinii* is the most active member of the AOD lesion microbiome, driving tissue maceration and host defence suppression. Furthermore, through assembly of draft genomes from metagenome datasets, we identified at least two bacterial taxa in the AOD microbiome that had not previously been identified, belonging to the *Clostridioides* and *Carnobacterium* genera, but exhibiting virulence-associated transcriptomic and proteomic activity in AOD lesions. This suggests that the AOD lesion microbiome is even more complicated than previously thought, and further research into the Gram-positive component is required.

## Results

### Functional potential of the AOD lesion microbiome and identification of two abundant Gram-positive bacteria through metagenome binning

**Fig. 1:**
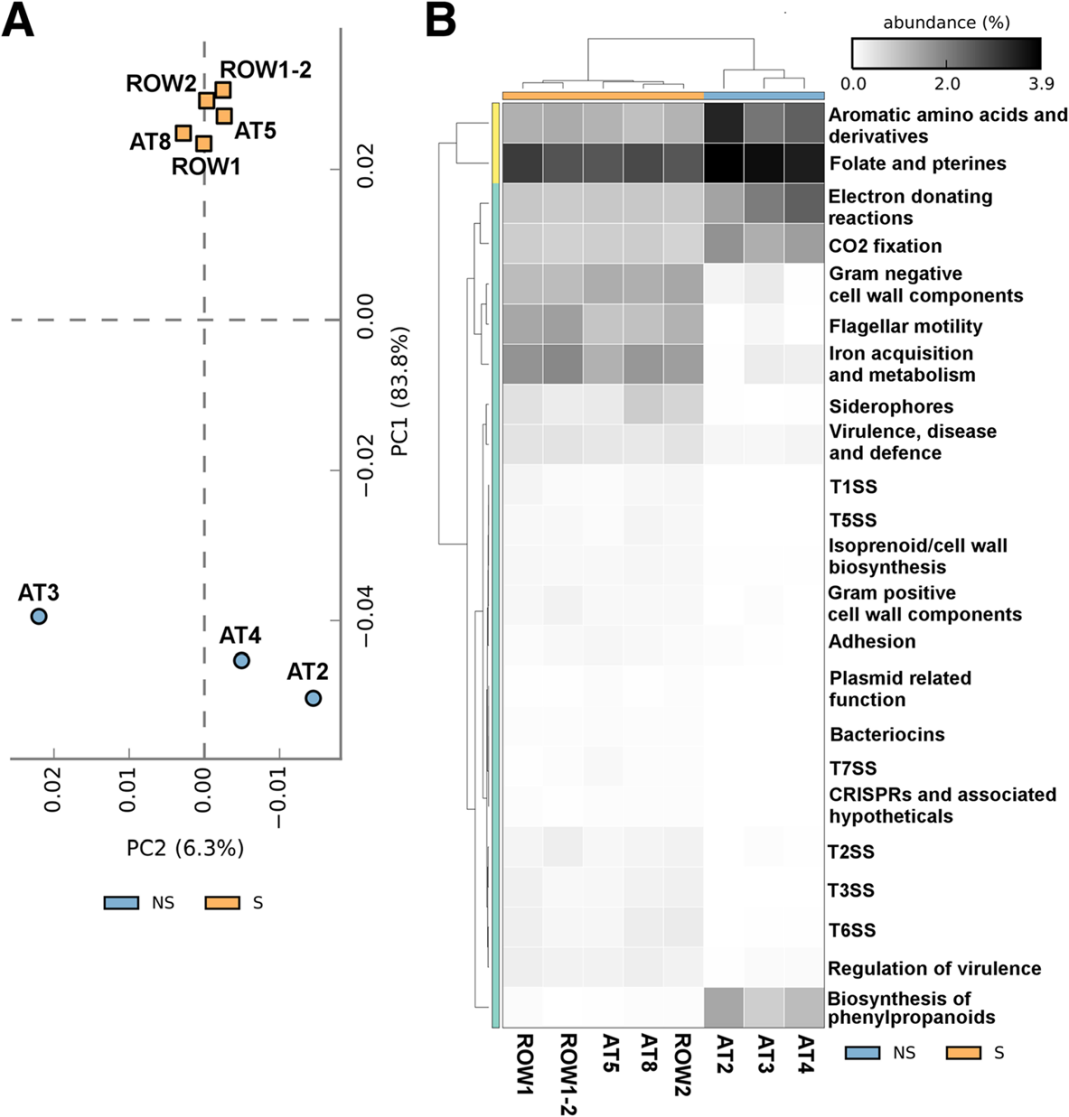
Metagenomics reveals distinct microbiomes and functional genes between symptomatic and non-symptomatic samples. **a** Shift of metagenomic functional categories relative abundances between symptomatic and non-symptomatic samples. The STAMP software was used to draw a heatmap comparing the relative abundances of level 2 SEED subsystems associated with bacterial virulence, plant defence and hormone production in the samples. **b** A principal component analysis of the samples based on functional SEED subsystem categories. SEED subsystem genesets were found to be statistically significant using White’s non-parametric *t* test and Benjamini-Hochberg multiple test correction

We have previously described the taxonomic composition of the AOD microbiome and the shift from the microbiome of healthy trees [4]. However, in order to identify the specific mechanistic processes that mediate lesion formation, a deeper functional analysis comparing the AOD microbiome and healthy microbiome was required. Thus, we aimed to identify categories of genes associated with AOD virulence and highlight the dramatic shifts taking place on a functional level. Samples were taken from three non-symptomatic *Q*. *robur* trees in Attingham Park, along with two symptomatic AOD *Q*. *robur* trees with drier lesions, and two symptomatic *Q. petraea* trees at Hill court park with fresher lesions where fluid was actively seeping from the lesions. As host (oak) DNA would dominate the sequencing libraries obtained using DNA extracted from host tissue, significantly reducing the microbiome signal, the host DNA was depleted using an NEB Next Microbiome enrichment kit in order to focus on the microbiota for this stage of the analysis. The functional potential of the microbiome was initially identified using MG-RAST [19] (Additional file 1: Table S1, Additional file 2: Table S2, Additional file 3: Table S3, Additional file 4: Table S4, Additional file 5: Table S5, Additional file 6: Table S6, Additional file 7: Table S7 and Additional file 8: Table S8), revealing that approximately 67–95% of the predicted genes in AOD lesion microbiomes were bacterial, whereas only 0.6–6% of genes in non-symptomatic samples were derived from bacteria. Functional microbiome analysis of SEED subsystem gene category abundances (functional gene categories) demonstrated a clear distinction between symptomatic and non-symptomatic samples, corroborating previous investigations of the AOD microbiome shift, and providing a genetic database for our holistic multi-omic analysis (Fig. 1a) [2–4, 6]. Specifically, the MG-RAST analysis demonstrated that functional SEED subsystem gene category abundances, associated with normal plant activity were significantly more abundant in non-symptomatic tree samples, while those associated with bacterial activity, bacteriophage activity and plant defence were more abundant in symptomatic trees (Additional file 9: Table S9, Fig. 1b).

The metagenome databases of annotated genes were combined revealing 627 distinct genes identified in all symptomatic samples, but undetected and thus considered absent in the non-symptomatic samples (Additional file 10: Table S10). These genes were 99% bacterial and included proteins involved in virulence (membrane transport, PCWDEs, iron scavenging, motility and chemotaxis, stress responses and regulation). In contrast, 223 distinct genes (80% plant associated, 20% fungal), involved mainly in general metabolism, were identified in all non-symptomatic samples, but in none of the symptomatic samples (Additional file 11: Table S11).

Although several new species of bacteria have been isolated from AOD lesions, it was important to attempt to identify any as-yet uncultivated microorganisms in the AOD lesion microbiome that may play an important role in lesion formation. Using metagenomic binning, we generated two draft genomes belonging to the genera *Clostridioides* and *Carnobacterium*, and other bacteria (Additional file 12: Table S12), from metagenomic reads, thus extending the AOD-associated microbiome of putatively unculturable organisms for further analysis. The predicted genome sizes were 5.45 and 2.42 Mbp and the genome completions were 97.5 and 66.7%, respectively for the *Clostridioides* and *Carnobacterium* bacteria, and both exhibiting < 5% contamination. However, as no ribosomal RNAs were identified in the *Clostridioides* genome, both genomes were determined to be medium quality drafts as outlined in the guidelines by Bowers et al. [20]. These genomes were annotated and found to contain PCWDE, secretion systems, catalases and ROS-defence associated genes, flagella and other virulence-associated genes (Additional file 12: Table S12 and Additional file 13). As a contrast to large public databases such as Swissprot where our newly identified bacterial species are not yet represented, the annotated genomes were combined into a narrowed-down database for specified bioinformatic analysis, containing the annotated oak transcriptome from NCBI (Additional file 14: Table S13), the annotated genomes of AOD-associated bacteria *B*. *goodwinii*, *G*. *quercinecans*, *R*. *victoriana* and other bacteria whose genomes had homology to AOD lesion metagenome coding domains; *Escherichia coli*, *Dickeya dadantii*, *Pectobacterium carotovorum*, *Erwinia billingae*, *Serratia marcescens* and *Clavibacter michiganensis* (Additional file 13). This database allowed us to specifically clarify the activity of each bacterial organism of interest in the AOD microbiome, as well as specify which host genes and proteins are active.

### Metatranscriptome analysis reveals active host defences and the primary active pathogens of the AOD microbiome

We performed metatranscriptome analysis of symptomatic and non-symptomatic inner bark tissue to identify the functional activity of the host and its microbiota and to specifically determine the function and relative role of *B*. *goodwinii*, *G*. *quercinecans*, *R*. *victoriana* and other bacteria in the AOD lesion microbiome. The metatranscriptomes were profiled and compared across samples (Additional file 15: Table S14, Additional file 16: Table S15, Additional file 17: Table S16, Additional file 18: Table S17, Additional file 19: Table S18, Additional file 20: Table S19 and Additional file 21: Table S20). In symptomatic tissue, 11–21% of annotated predicted transcripts were bacterial (2–3% in non-symptomatic tissue) (Additional file 15: Table S14, Additional file 16: Table S15, Additional file 17: Table S16, Additional file 18: Table S17, Additional file 19: Table S18, Additional file 20: Table S19 and Additional file 21: Table S20). These data demonstrate that the genetic shift in the metagenome also translates into an enhanced functional activity of bacteria.

We identified 216 transcripts present in all AOD symptomatic samples but absent in non-symptomatic trees (based on annotation, Additional file 22: Table S21). Of the symptomatic transcripts, 94% were associated with bacteria, partly involved in virulence (regulation, membrane transport, signalling, biofilm, sporulation and PCWDEs), the remaining 6% were associated partly with plant defence and cell wall synthesis, revealing increased bacterial phytopathogenic activity in symptomatic tissue. Conversely, 263 transcripts were identified across all non-symptomatic samples, but not in any symptomatic sample (97% eukaryotic, 3% bacterial, Additional file 23: Table S22).

**Fig. 2:**
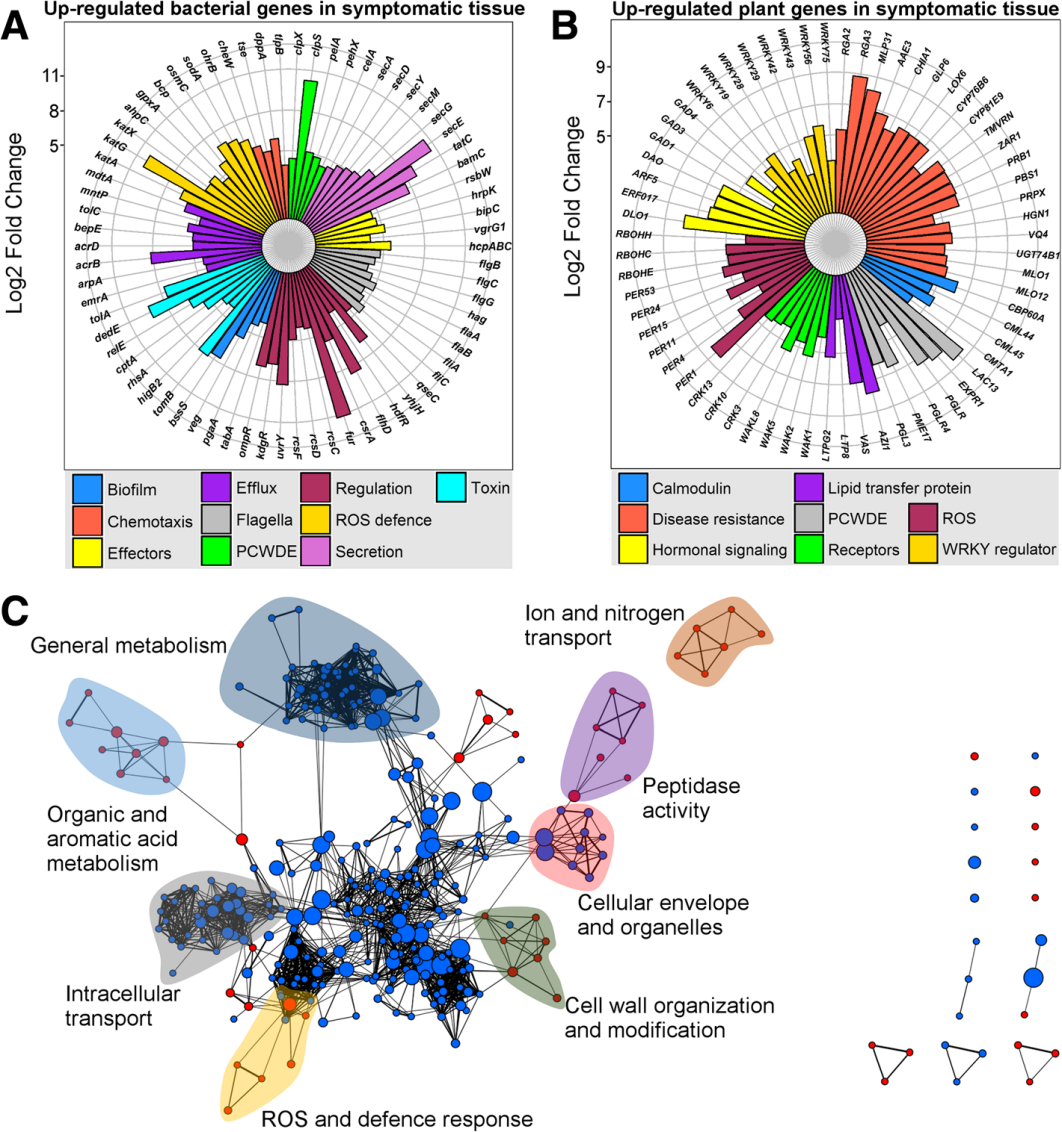
Metatranscriptomics reveals distinct gene expression profiles between symptomatic and non-symptomatic samples. **a** Bacterial virulence-associated genes significantly upregulated in symptomatic samples compared to non-symptomatic samples. Genes were determined to be significantly different in expression by using the limma package in R (statistical cutoff at FDR < 0.05, and log_2_ cutoff at < − 2 and > 2). Genes were separated into different colour-coded categories. **b** Oak tree defence-associated genes, based on the Swissprot database, upregulated in symptomatic samples compared to non-symptomatic samples. Genes were identified the same way as in **a** and separated into different categories as colour coded in the figure. Circles in light grey outgoing from the centre indicate the different log_2_ fold changes, with respective numbers (5, 8, 11 in **a** and 5, 7, 9 in **b**) indicated to the side of the plots. **c** Geneset Enrichment Analysis highlights the global impact on genesets of infected oak tree cells. Genes with closest homology to Arabidopsis, determined to be statistically significantly altered in expression in symptomatic samples compared to non-symptomatic samples, were used in a geneset enrichment analysis (GSEA). Depicted in the figure are genesets found in the GO and KEGG databases. The figure was made using the GSEA results in the Enrichment Map plugin (Bader Lab) for the Cytoscape software (version 3.2). Red circles signify upregulated genesets, and blue circles signify downregulated genesets

In order to generally assess quantitative differences between non-symptomatic and symptomatic metatranscriptomes, we performed a gene expression analysis, identifying 6419 differentially expressed genes in symptomatic tissue (Additional file 24: Table S23). The 3064 downregulated genes in symptomatic tissue mainly displayed homology to plants (69%), fungi (16%), animals (9%) and bacteria (5%), while the 3355 upregulated (upregulated is used generally here to include bacterial genes that may for example be unrepresented in non-symptomatic tissue) displayed homology to bacteria (61%), plants (32%), animals (4%) and fungi (2%). Many of the upregulated bacterial genes were associated with virulence, for example, biofilm formation, chemotaxis and motility, effectors, efflux pumps, PCWDEs, virulence regulation, reactive oxygen species (ROS) defence, protein secretion systems and toxin production (Fig. 2a). Upregulated plant genes associated with symptomatic trees included calmodulin binding and production, disease resistance, hormonal signalling, PCWDEs, membrane receptors, ROS production and protection and the WRKY superfamily stress response transcription factors (Fig. 2b). Furthermore, 31 downregulated and 66 upregulated genes were identified as originating from viruses. The majority of downregulated viral genes belonged to plant viruses, while the majority of upregulated viral genes belonged to bacteriophages (Additional file 24: Table S23).

To further clarify the host response in the symptomatic tissue, we performed a geneset enrichment analysis (GSEA) on the expression data homologous to Arabidopsis genes (Fig. 2c) [21, 22]. GSEA demonstrated upregulation of 190 genesets and downregulation of 276 genesets (Additional file 25: Table S24). Upregulated genesets were partly associated with defence and wounding response, ROS burst, cell wall modification and cell death. Downregulated genesets were partly associated with organelle organisation, circadian rhythm, protein transport, cell-cell signalling, photoperiodism and regulation of development, demonstrating a redistribution of resources in the host from growth to defence.

We developed a narrowed-down database containing bacteria identified as abundant and important in AOD lesion formation in our previous study [4], along with bacterial draft genomes extracted from the metagenomic data, and the *Q*. *robur* transcriptome available in the NCBI database, which we annotated (Additional file 14: Table S13) [23]. The narrowed-down database allowed for specifying the representation of all bacteria of interest and the oak host in AOD lesions the metatranscriptomes and metaproteomes, and comparing symptomatic against non-symptomatic field samples (Additional file 26: Table S25, Additional file 27: Table S26, Additional file 28: Table S27, Additional file 29: Table S28, Additional file 30: Table S29, Additional file 31: Table S30 and Additional file 32: Table S31). We could significantly detect (FDR < 0.05, fold change > 2) 499 upregulated genes in symptomatic samples and 92 downregulated genes. Of the 499 upregulated genes, 295 were *B*. *goodwinii* genes, 198 were oak genes and 6 belonged to the predicted bacterium of the genus *Clostridioides* (Additional file 33: Table S32). However, studying the identified gene transcripts of each sample, we could detect PCWDEs (for example oligogalacturonate lyases, proteases, pectate lyases, pectate disaccharide lyases and cellulases), toxins (for example toxin A, toxin RTX, HigB-2 and Colicin V) and other virulence-associated genes (for example effectors HopM1, YopJ, flagella and pathogenicity factors) as active (Additional file 26: Table S25, Additional file 27: Table S26, Additional file 28: Table S27, Additional file 29: Table S28, Additional file 30: Table S29, Additional file 31: Table S30, Additional file 32: Table S31 and Additional file 33: Table S32). The genes of interest to virulence and survival were mainly identified as belonging to *B*. *goodwinii* (Additional file 34: Figure S1A) but also to the putative *Clostridioides* and *Carnobacterium*. Using our narrowed-down database, the percentage of bacterial reads in the metatranscriptome of AOD symptomatic samples ranged between 1.1 and 3.3%, with *B*. *goodwinii* corresponding to 33–82% of those reads. In non-symptomatic samples, the percentage of bacterial reads was only 0.02–0.07%, with no clear difference between different species. For the host, we could identify genes covering similar categories as for the Swissprot analysis (Additional file 34: Figure S1B), with several defence-associated regulators and genes activated, such as mitogen-activated protein kinase, kinase *AtM2K9*, suggesting activation of the mitogen-activated protein kinase stress response cascade. Furthermore, the defence-associated genes *AtNDR/HIN1*-like protein 3 and 13 were significantly upregulated suggesting enhanced defence, along with genes for toxin and ROS resistance such as *AtGSTU8* and *AtDTX29*.

### Metaproteome analysis suggests *B*. *goodwinii* is the dominant pathogen in the AOD lesion microbiome

**Fig. 3:**
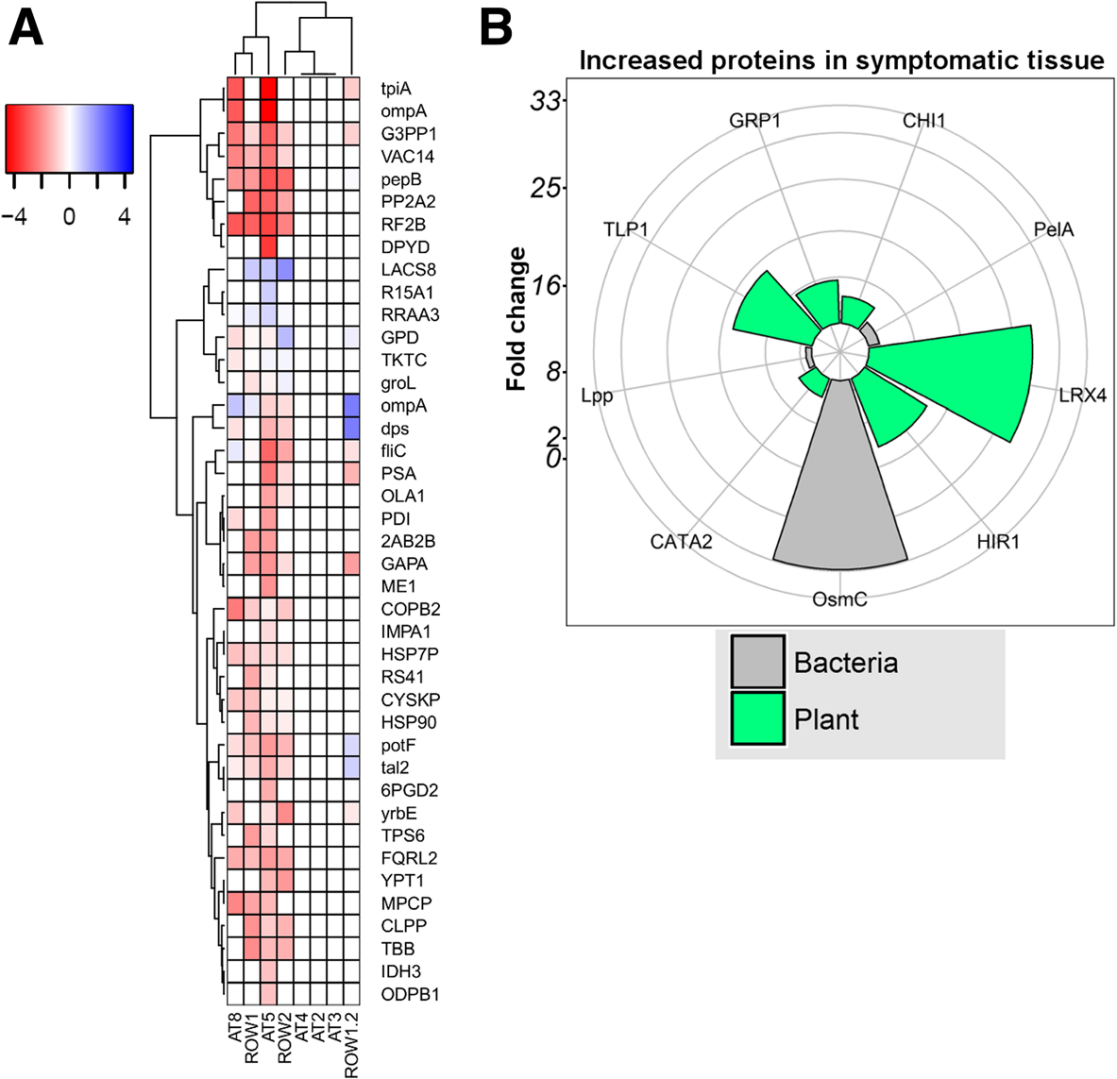
Metaproteomics reveals protein abundance profiling between symptomatic and non-symptomatic samples. **a** Bacterial and host defence-associated proteins detected in AOD symptomatic tissue. Heatmap of MAD-scaled log_10_ counts for proteins detected in symptomatic samples but not detected in non-symptomatic samples. The colour key depicts the log_10_ MAD scale of protein counts in the different symptomatic samples. **b** Pathogenic and host defence-associated proteins differently abundant in symptomatic samples, depicting the increase of bacterial virulence-associated proteins in symptomatic tissue compared to non-symptomatic. Proteins were determined as significant using a *t* test on MAD normalised proteins of interest. Circles in light grey going out from the centre indicate the different log_2_ fold changes, with the numbers indicated to the side of the plots

In order to further confirm the results of the metagenomic and metatranscriptomic data, by identifying active proteins of the microbiome members of interest to the AOD disease aetiology, and the host defence, we performed metaproteomic analysis using mass spectrometry. Using genes identified via metagenomics and metatranscriptomics as a reference database, we identified 629 proteins, of which 59 were bacterial and 570 eukaryotic (Additional file 35: Table S33). As expected, bacterial proteins were detected in all tissue samples but were generally higher in abundance in symptomatic samples. However, several core proteins of phytopathogenic bacteria were detected only in symptomatic tree samples, such as FliC, PepB, PotF and OmpA, along with various plant-associated transketolases, alcohol dehydrogenases and aldolases (Fig. 3a). In a comparative analysis of symptomatic versus non-symptomatic samples, bacterial proteins PelA (plant cell wall-degrading enzyme) and OsmC (survival) were significantly increased in symptomatic tissue (Fig. 3b), along with host-associated proteins CHI1 (chitinase), CAT2 (catalase), LRX4 (cell wall formation regulation), HIR1 (hypersensitive reaction) and GRP1 and TLP1 (lignin formation). Metaproteomics clearly demonstrates an increased abundance of bacterial proteins, and a host defence response, in AOD lesions.

Using the narrowed-down database described for the metatranscriptomics, we re-performed the metaproteome analysis (Additional file 36: Table S34). We found a total of 122 proteins significantly (*p* < 0.05, fold change > 2) differently abundant in symptomatic samples compared to non-symptomatic samples using student’s *T* test, or undetected in all non-symptomatic samples and detected in all symptomatic samples (Additional file 37: Table S35). Additionally, a Benjamini-Hochberg multiple test correction was performed on the *p* values, which yielded significance (FDR < 0.05) for one gene, BG_04787, a 4-hydroxy-tetrahydrodipicolinate synthase. However, the identified proteins in general supported the metagenomic and metatranscriptomic results. Among the proteins with increased abundance in AOD symptomatic samples were those of *B*. *goodwinii* and *G*. *quercinecans*, as well as that of our predicted *Clostridioides*. Only one protein, predicted from oak transcript DN950644.1, homologous to a vesicle transport protein found in *Arabidopsis thaliana*, was increased in abundance in healthy samples; the other 121 proteins were more abundant in symptomatic samples, of which 23.5% were bacterial. Of those proteins, 18 proteins belonging to *B*. *goodwinii* were detected, including a superoxide dismutase, aldolases, a catalase and lipoproteins, suggesting survival in the oak host. Several host pectinesterases, proteases and chitinases were increased in abundance, suggesting PCWDE maceration taking place and anti-fungal activity. Furthermore, 3 proteins belonging to *G*. *quercinecans* were significantly increased in symptomatic tissue, including a Colicin V secretion protein, along with a signal recognition protein and a hypothetical protein of unknown functions, suggesting bacterial competition. The data of the narrowed-down database here provides a similar but re-focused picture of bacterial survival and virulence from the most prominent AOD microbiome member, *B*. *goodwinii*, along with host defence in the AOD lesions (Additional file 38: Figure S2).

## Discussion

AOD is a complex and rapidly expanding decline-disease within the broader oak decline complex, currently representing a significant threat to native oak in England and Wales [2]. Although the taxonomic questions regarding the causes of AOD have been addressed, an in-depth investigation into the underlying functional mechanisms that trigger and mediate lesion formation has been lacking [4]. We aimed to investigate the disease on a molecular genetic level and present a powerful combination of methodologies to approach plant diseases. Here, our integrated multi-omic analysis has provided novel insights into stem microbiome activity and tree defence responses, extending current research of polymicrobial diseases in plants. The host DNA was depleted in extracts for microbiome-targeted analysis, while host transcripts and proteins were retained in RNA and protein extracts for simultaneous host and microbiome activity analysis. This approach may explain the greater overlap of metatranscriptome and metaproteome data between different samples (Additional file 39: Table S36). The detection of predominantly plant-associated genes in metagenome datasets from the non-symptomatic samples suggests that the depletion of host DNA was not complete. However, while the microbiome enrichment step depletes the host DNA, it does not completely remove all host DNA. In addition, both culture-based and molecular studies have suggested a very low proportion of microbial biomass in healthy tissue, which makes it reasonable to suggest that even with a microbiome enrichment step, a large proportion of host DNA will likely remain [4, 24].

The increased bacterial activity in AOD lesions may explain an increased bacteriophage activity, as bacteriophages capitalise on the abundant presence of hosts (i.e. bacteria) and stressful conditions, as suggested in bacteriophage predation studies [25]. We previously demonstrated in our taxonomic study that fungi, animals (aside from *A*. *biguttatus*) and viruses were not significantly associated with AOD [4]. Furthermore, bacterial and plant-associated genes were detected as present and consistently active across the -omic datasets presented here, further corroborating our previous study of the importance of these aspects. Although *A*. *biguttatus* is still an interesting agent associated with AOD, further studies are required to determine its role, as proteins and genes associated with beetles or insects were not consistently found here across our datasets.

The study was conducted in field conditions, where we located AOD in two different locations, and in two different species of oak (*Q*. *robur* and *Q*. *petraea*). The samples AT5 and AT8 were from lesions exhibiting drier, callused later stages of AOD, while the samples ROW1, ROW1-2 and ROW2 were taken from trees exhibiting fresh wet lesion areas. The differences in tree and location may impact statistical analyses, along with extraction efficiency differences in macerated lesion tissue and healthy bark. Macerated tissue is already degraded to an extent, but it also contains more phenolics, DNAses, RNAses and proteases and other compounds inhibiting the extraction efficiency. However, overall the data collected here across the -omic disciplines exhibits strong similarities for the symptomatic samples in terms of microbiome community structure, and transcriptomic and proteomic analysis results, providing a strong framework for the functional mechanissms of AOD.

The metatranscriptomic data highlights substantial host-microbiome interactions, demonstrating an increase in transcripts of phytopathogenic bacteria in stem lesions along with transcripts involved in host defence. The redundancy in many of the bacterial transcripts (multiple differently assembled transcripts annotated as the same gene, even housekeeping genes) further suggests that different bacteria are operating as a plant tissue macerating community. The coincidental up- and downregulation of plant transcripts classified as the same gene, or within the same functional category (e.g. defence, regulation and hormone signalling), demonstrates complex host activity. This activity is clarified by the GSEA, highlighting a transcription profile in symptomatic oak trees general to microbial pathogenic infection; the triggering of defence-associated processes in the plant cells, balanced by the downregulation of development, internal organisation and metabolism [26]. Identified bacterial virulence-associated genes might be co-opted for tree diagnostics and monitoring, whereas beneficial plant regulators and pathogen defence genes might be used as markers for future oak breeding and monitoring.

**Fig. 4:**
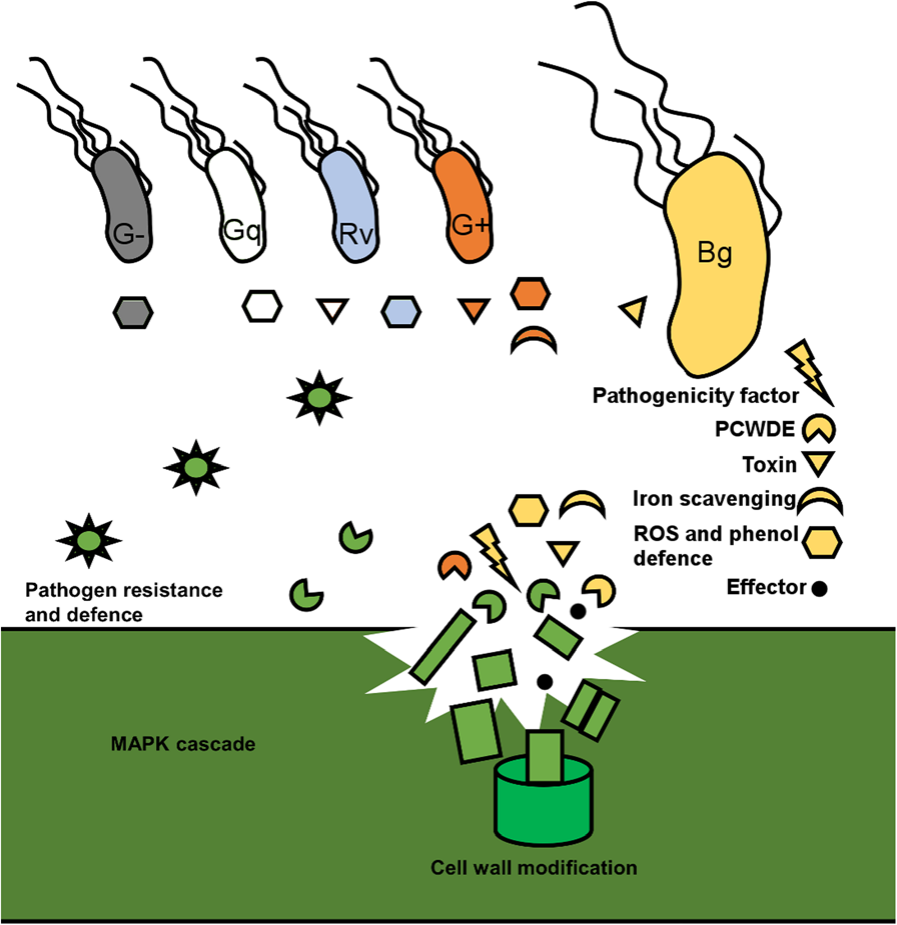
Multi-omic profiling provides an overview of the different -omics and models of the interactions between host and pathogens. Using our in-house narrowed-down database, *Brenneria goodwinii* was found to be the most active bacterium and the primary pathogenic agent in the AOD lesion microbiome based on metatranscriptomics and metaproteomics (depicted as Bg and yellow colour in the figure). *Gibbsiella quercinecans* (Gq and white colour in the figure) and *Rahnella victoriana* (Rv and blue colour in the figure) were found to provide some ambiguous activity. Other Gram-negative species (collectively called G−, with grey colour in the figure) and two predicted Gram-positive bacterial genomes (collectively called G+ in the figure and coloured red) provided some virulent activity as well. The size of each bacterial species/group indicates their relative abundance in each dataset (not to scale). The colour of active groups of virulence-associated genes of interest are coloured coded as their associated bacterial origin. Active plant proteins and genes are coloured dark green. The depicted shapes correspond to different virulence-associated functions

Our results reveal the details of the microbiome activity in the AOD lesions, on one hand by using a broad-spectrum public database such as Swissprot (Additional file 1: Table S1, Additional file 2: Table S2, Additional file 3: Table S3, Additional file 4: Table S4, Additional file 5: Table S5, Additional file 6: Table S6, Additional file 7: Table S7, Additional file 8: Table S8, Additional file 9: Table S9, Additional file 10: Table S10 and Additional file 11: Table S11; Additional file 14: Table S13, Additional file 15: Table S14, Additional file 16: Table S15, Additional file 17: Table S16, Additional file 18: Table S17, Additional file 19: Table S18, Additional file 20: Table S19, Additional file 21: Table S20, Additional file 22: Table S21, Additional file 23: Table S22, Additional file 24: Table S23, Additional file 25: Table S24 and Additional file 26: Table S25), and on the other a narrowed-down database containing bacteria of interest and gene transcripts identified in oak. Specifically, we observed the dominance of a *B*. *goodwinii* in virulence-associated activity, aided by Gram-positive and Gram-negative bacteria of interest and concomitant detection of host defence responses typical of phytopathogen infection in oak stems. The analysis confirms an active enterobacterial and pectobacterial community displaying active virulence factors not detected in healthy trees. Previous taxonomic analysis of the AOD microbiome suggested that there is an AOD microbiome composed of several bacteria in AOD lesions, where *B*. *goodwinii* is the most abundant bacterium [4]. Our functional metatranscriptomic and metaproteomic analysis corroborates these data, showing that in numbers of virulence-associated genes and amount of transcripts (Additional file 25: Table S24, Additional file 26: Table S25, Additional file 27: Table S26, Additional file 28: Table S27, Additional file 29: Table S28, Additional file 30: Table S29, Additional file 31: Table S30, Additional file 32: Table S31, Additional file 33: Table S32, Additional file 35: Table S33, Additional file 36: Table S34 and Additional file 37: Table S35), *B*. *goodwinii* is the most active member of the AOD microbiome based on our metaproteome and metatranscriptome data (Fig. 4, Table 1), followed by *G*. *quercinecans* and *R*. *victoriana* and drafted genomes assigned to Gram-positive bacteria *Clostridioides* and *Carnobacterium*. These Gram-positive bacteria may previously have evaded interest due to a lack of isolation from lesions, and because prior to draft genome assembly in this analysis, they were not represented in public databases. Consequently, these data demonstrate the significant potential of draft genome assembly from metagenome datasets to identify candidate microorganisms for targeted analysis.

**Table 1:** List of genes and proteins of interest involved in virulence, bacterial survival and interactions identified in the metaproteome and metatranscriptome of AOD lesions and their corresponding bacterial species/group of origin

Gene annotation	*B*. *goodwinii*	*G*. *quercinecans*	*R*. *victoriana*	G+	G−
Antitoxin HicB	T				
Bacterial type II secretion system protein F domain protein	T				
Protease CtpB				T	
Catalase	PT	T		T	T
Colicin V secretion protein CvaA	T	P			
CRISPR-associated protein Csy3		T			
CsrB	T	T	T		T
Cysteine protease avirulence protein AvrPphB	T				
Effector protein HopM1	T				
Effector protein YopJ	T				
Entericidin B membrane lipoprotein	P				
Flavodoxin	PT				
Harpin HrpN	T				
HTH-type transcriptional regulator KdgR	T				
Iron-binding protein IscA	T				
Multidrug efflux pump subunit AcrB	T		T		T
Oligoendopeptidase F, plasmid				PT	
Oligogalacturonate lyase	T				
Oligopeptidase A	T				
Pathogenicity factor	T				
Pectate lyase A precursor	T				
Peroxiredoxin OsmC	PT				
Persistence and stress-resistance toxin PasT	T				
ProP effector	T				
Protease 2	T				
Protease HtpX	T				
Putative oxidoreductase SadH		T			
Putative type II secretion system protein E	T			T	
Response regulator UvrY	T				
Serine protease AprX				T	
Superoxide dismutase [Mn]	PT			T	
Thermostable beta-glucosidase B	T			T	
Toxin A				PT	
Toxin B				T	
Toxin HigB-2	T				
Toxin-antitoxin biofilm protein TabA	T				
Type II secretion system protein D	T				PT
Type IV secretion system protein VirB		T			
Virulence factor SrfB	T				
Virulence regulon transcriptional activator VirF	T				
Virulence sensor histidine kinase PhoQ	T				

G+ signifies the two Gram-positive bacteria belonging to the *Clostridioides* and *Carnobacterium*, while G− signifies other Gram-negative bacteria excluding *B*. *goodwinii*, *G*. *quercinecans* and *R*. *victoriana.* P signifies detection of that gene in the proteomic data, while T signifies detection of the gene in the transcriptomic data from AOD lesions

Taking the metatranscriptomic and metaproteomic data together, the results demonstrate that at least *B*. *goodwinii* and possibly other bacteria from the AOD microbiome are also present in healthy tissue, although at very low rates, and without significant virulence-associated activity. However, the presence of low levels of *B*. *goodwinii* in non-symptomatic trees may represent another example of the established phenomena that certain members of the microbiome may represent pathobionts; temporarily benign microorganisms that under altered conditions in the host may impart disease causation, and often, their activity is triggered or regulated by other members of the microbiota [17]. This phenomenon has also been described for the olive knot disease where activity of the key pathogen *Pseudomonas savastanoi* is regulated by the non-pathogenic *Erwinia oleae*, *E. toletanta* and *Pantoea agglomerans* [27]. Thus, *B*. *goodwinii*, *G*. *quercinecans*, *R*. *victoriana* and other bacteria identified in healthy tissue may perform this type of role. Comparing the output of metatranscriptomics and metagenomics using a generalised database such as Swissprot with the narrowed-down databased created for this study, there was a strong similarity in the shift in bacterial activity between symptomatic and non-symptomatic samples. With the narrowed-down database, we can through proteomic and transcriptomic analysis speculate that *B*. *goodwinii* is primarily acting in a survivalist manner in mature AOD lesions, utilising catalases and superoxide dismutases as a way to combat the host defences, and using entericidin for inhibiting other bacteria. However, samples ROW1 and ROW1-2, which were taken from trees with significantly more active lesions (fresh and wet bleeding, instead of dry “caked over” scab-like lesions in AT5 and AT8) also reveal a stronger activity in PCWDE and virulence-associated genes (regardless of database). The Gram-positive bacteria of this study were also found to be active primarily in the fresher lesions, while almost absent in the older lesions. Consequently, these results suggest that the Gram-positive bacteria lead a transitory phase, while *B*. *goodwinii* is highly active during maturity of the AOD lesions. The other Gram-negative bacteria involved in the AOD lesions were found to perform mainly interbacterial actions and stress responses. These results suggest that *G*. *quercinecans*, *R. victoriana* and other Gram-negative bacteria, at the advanced stages of the AOD lesions sampled here, are aiding the more virulent activities by *B*. *goodwinii* and the Gram-positive bacteria by supressing the effects of the oxidative and antimicrobial environment in the lesion while at the same time competing with each other. The tree host seems to have several of its own PCWDEs active in the AOD lesions, which may be of detriment to the host’s own health. Activity of tree chitinase in AOD may explain the absence of any fungal organisms in the AOD microbiome [28, 29]. The results here also demonstrate similar general trends on a metaproteomic and metatranscriptomic level regardless of type of bioinformatic tools used. The databases used in future multi-omic analyses will prove more fine-tuned and reliable as the publicly available oak genome and general microbe genome databases are further refined and annotated.

## Conclusions

Multi-omic analysis of oak-microbiome interactions in non-symptomatic and AOD symptomatic trees has provided important insights into the oak hologenome and its role in health and disease. This study demonstrates the power of draft genome reconstruction and multi-omicanalyses for characterising microbiome activity and interactions and concomitant host defences, providing a knowledge base and conceptual framework that will be increasingly important in future studies of host-microbiome interactions [13, 30], and in the management of AOD, that represents a significant threat to the UK’s iconic oak.

## Methods

### In-field oak tissue sampling

Collection of inner bark tissue from oak stems was performed in June 2015. The appropriate permissions were obtained from land owners prior to sampling, and Forest Research guidelines were followed for sampling. Briefly, the fully barked panels (approximately 10 × 15 cm WxB) were removed from the bleeding points of affected trees including the lesion margin and surrounding visually unaffected tissue (or in non-symptomatic trees, from positions of similar above-ground height) using a sterilised mallet and chisel. The sampled tissue was immediately flash frozen on dry ice. In total, 8 samples were extracted from 7 trees; 3 non-symptomatic (samples AT2, AT3 and AT4) and two symptomatic (samples AT5 and AT8) from English oak (*Q*. *robur*) at Attingham Park, and two symptomatic (samples ROW1, ROW1-2, each sampled from different bleeds on the same tree, and ROW2) from sessile oak *(Q*. *petraea)* in Hill Court near Ross-on-Wye (see Denman et al., [4] for further details on field sites and sampling methods). In the laboratory tissue pieces from the frozen, active lesion margin were removed using a chisel and mallet and homogenised by grinding with a sterile mortar and pestle. DNA, RNA and protein were extracted from the homogenised tissue of each sample (see below), aside from sample ROW2 not providing RNA of sufficient quality and quantity.

### DNA extraction

DNA was extracted from approximately 50 mg of homogenised oak tissue, using the DNeasy Plant Mini kit (Qiagen) according to the manufacturer’s instructions. Quality and concentration of samples were determined using agarose gel electrophoresis and the Qubit dsDNA HS assay kit (Thermo Fisher) according to the manufacturer’s instructions. In order to enrich microbiome DNA, the host DNA was depleted from the sample using the NEBnext microbiome DNA enrichment kit (New England Biolabs) according to the manufacturer’s instructions. Subsequently, the DNA was purified and concentrated using the Genomic DNA Clean and Concentrator kit (Zymo Research) according to the manufacturer’s instructions and stored at − 20 °C.

### RNA extraction

RNA was extracted from approximately 50 mg of homogenised oak tissue using a modified procedure for isolating RNA from woody plant material [31]. Briefly, 5 ml of extraction buffer (4 M guanidine thiocyanate, 0.2 M sodium acetate pH 5.0, 25 mM EDTA, 2.5% (*w*/*v*) polyvinylpyrrolidone and 1% (*v*/*v*) β-mercaptoethanol) was added to oak tissue kept frozen in a sterilised mortar using liquid nitrogen. The frozen tissue in extraction buffer was further ground until thawed. Subsequently, an additional 2.5 ml of extraction buffer and 500 μl of 20% sodium lauroyl sarcosinate were mixed into the sample. The sample mixture was shaken vigorously at room temperature for 15 min and further processed using the RNeasy Plant Mini kit (Qiagen). After centrifugation in the QIAShredder column, 350 μl of the supernatant was mixed with 0.9 volumes of ethanol and subsequently centrifuged in the RNeasy Mini column. After this centrifugation step, the manufacturer’s instructions for the RNeasy Plant Mini kit were followed. The extracted RNA was treated with DNase I (Qiagen) and further concentrated and purified using the RNeasy MinElute Cleanup kit (Qiagen) following the manufacturer’s instructions. The purified RNA was checked for quality using 1% agarose gel electrophoresis and a NanoDrop spectrophotometer (LabTech), and the concentration determined using the Qubit RNA HS assay kit (Thermo Fisher) following the manufacturer’s instructions. Subsequently, rRNA was depleted from RNA extracts using a 1:1 combination of the Ribo-Zero rRNA Removal kits for plant seed/root and for bacteria (Illumina) according to the manufacturer’s instructions. The rRNA-depleted samples were again purified using the RNeasy MinElute Cleanup kit (Qiagen) again and stored at − 80 °C.

### Protein extraction

Proteins were extracted from approximately 50 mg of homogenised oak tissue using a modified method for protein extraction from woody tissue [32]. Briefly, the oak tissue was ground in 2-ml solubilisation buffer (50 mM Tris-HCl, 25 mM EDTA, 500 mM thiourea, 0.5% DTT). The mixture underwent shaking (150 rpm) for 1 h at ambient temperature. The samples were subsequently centrifuged at 20000*g* for 20 min, and the supernatant was extracted and stored at 4 °C. The procedure was repeated using the remaining pellet. The supernatant was extracted and pooled with the previous supernatant. Ice cold 20% trichloric acid in acetone with 0.5% DTT was added in a 1:1 ratio to the supernatant pool and precipitated at − 20 °C overnight. After precipitation, the mixture was centrifuged at 20000*g* for 60 min and washed with ice cold acetone (centrifuged at 20000*g* for 30 min). The pellet was air dried, re-suspended in 3% SDS solution and stored at − 80 °C.

### Metagenomic analysis

DNA samples were sent to the Centre for Genomic Research (CGR) at the University of Liverpool for sequencing. Samples were assayed for quality using a Fragment Analyzer (Advanced Analytical Technologies). Libraries were prepared using the Nextera XT Library Preparation kit (Illumina), and subsequently paired-end sequenced (2 × 125 bp) on one lane of the Illumina HiSeq platform. Raw sequences were trimmed using Cutadapt 1.2.1 and additionally Sickle 1.200 [33–35]. The total size of metagenome data was 52 gigabases (Gb).

For functional analysis, the trimmed reads were assembled de novo using Ray Meta (version 2.3.1) [36]. A k-mer size of 51 was used for the assembly of all sample datasets (N50 of 896-1116 for non-symptomatic samples, and N50 of 6154-127666 for symptomatic samples). Functional analysis was performed on the assembled contigs using the on-line metagenome analysis platform MG-RAST, which provided abundances in SEED subsystems for the samples. These subsystems were compared between symptomatic and non-symptomatic samples using the STAMP software package (version 2.1.3) [37]. SEED subsystems with a significant difference (FDR < 0.05) between symptomatic and non-symptomatic samples were identified using STAMP for performing a White’s non-parametric *t* test and Benjamini-Hochberg multiple test correction.

In order to detect bacteria of potential interest that may have evaded detection from traditional isolation methods that have been performed previously in AOD research, we utilised MetaBAT v0.32.4 to construct putative genomes from the metagenomic data [38]. The binned genomes were further analysed using CheckM v1.0.6 and AMPHORA2 to determine their taxonomy [39, 40]. Gene prediction and annotation of the binned genomes was performed using Prokka v1.11 [41].

### Metatranscriptomic analysis

RNA samples were sequenced by the CGR, Liverpool. Samples were further assayed for quality using an Agilent 2100 Bioanalyzer and the Eukaryote Total RNA Pico Series II. Libraries were prepared using the strand-specific ScriptSeq kit (Illumina), and subsequently paired-end sequenced (2 × 125 bp) on one lane of the Illumina HiSeq platform. Raw sequences were trimmed using Cutadapt 1.2.1 and additionally Sickle 1.200. The total size of the metatranscriptome sample data was 54 Gb.

Trimmed high-quality sequences were assembled de novo using Trinity (version 2.2.0) [42, 43]. The Trinonate (version 3.0.0) pipeline combined with bowtie (version 1.1.2) and RSEM (version 1.2.29) were used to functionally annotate Trinity contigs using the Swissprot database (based on simultaneous hits using both BLAST-P *e* value < 10^− 5^ and BLAST-X *e* value < 10^− 20^) and to align reads and quantify transcripts [44, 45]. Putative rRNA sequences were identified using RNAmmer (version 1.2) [46].

For differential expression analysis, raw paired reads from all metatranscriptomes were combined and analysed using the Trinonate pipeline as described. Predicted bowtie reads and RSEM-based counts were used in combination with the voom function of the limma R package to determine significantly differentially expressed genes between non-symptomatic and symptomatic samples (cutoff FDR < 0.05 and a minimal log_2_ fold change < − 2 and > 2) [47, 48].

Geneset enrichment analysis (GSEA) was performed on Arabidopsis-associated genes demonstrating a statistically significant change in expression as determined by the voom analysis. Geneset enrichment analysis allows for an overview of the impact of relative gene expression on larger processes (genesets). This is based on a Kolmogorov-Smirnov statistics of highly expressed genes between two traits, which has been used based on statistically significant expression profiling results of RNA sequencing data [22, 49, 50]. Genesets used for analysis were provided in the PlantGSEA platform database [21]. This database incorporates databases from the Gene Ontology Consortium (GO) and the Kyoto Encyclopedia of Genes and Genomes (KEGG) among others [51–54]. The analysis was performed on the GenePattern server (Broad Institute). Software parameters included 1000 permutations and a gene set size range from 5 to 1000 [22, 55, 56]. A *P* value cutoff of < 0.05 using a GSEA Kolmogorov-Smirnov-based statistics was set to determine genesets with significantly altered expression.

We previously identified three primary bacterial species as causal agents of AOD lesion formation, along with several other bacteria associated with a microbiome shift. In order to determine which bacteria and bacterial genes are truly active in lesions, we compiled a narrowed-down database based on the 10 most abundant bacterial species in AOD lesions as determined by Kraken analysis and 16 of the most complete predicted genomes from the metagenomic data. Furthermore, we added the oak transcriptome based on the NCBI database proposed by Ueno et al. [23], which we annotated using the Trinotate pipeline with Swissprot as a reference (Additional file 12: Table S12). The database was created as the bacterial species we are certain are causing AOD are poorly represented in databases such as Swissprot, which on the other hand has the advantage of containing more data. Thus, this narrowed-down database was used as an alternative reference for read alignment of the metatranscriptomes using Bowtie2 v1.1.2 [57], and transcripts were counted for each sample using eXpress v1.5.1 [58], and a cutoff of at least 3 counted transcripts per sample was used. Expression profiling between sample groups was performed as previously described using voom, using a cutoff of FDR < 0.05 and a fold change > 2.

### Metaproteomic analysis

Protein samples were sent to the Proteomics Facility at the University of Bristol for analysis using mass spectrometry. Aliquots of eight samples were digested with trypsin (2.5 μg trypsin per 100 μg protein; 37 °C, overnight), labelled with Tandem Mass Tag (TMT) ten plex reagents according to the manufacturer’s protocol (Thermo Fisher Scientific) and the labelled samples pooled. The pooled sample was fractionated by high pH reversed-phase chromatography using an Ultimate 3000 liquid chromatography system (Thermo Fisher Scientific). The resulting fractions were evaporated to dryness and re-suspended in 1% formic acid prior to analysis by nano-LC MSMS using an Orbitrap Fusion Tribrid mass spectrometer (Thermo Scientific). High pH RP fractions were further fractionated using an Ultimate 3000 nanoHPLC system in line with an Orbitrap Fusion Tribrid mass spectrometer (Thermo Scientific). Peptides were ionised by nano-electrospray ionisation at 2.0 kV using a stainless steel emitter with an internal diameter of 30 μm (Thermo Scientific) and a capillary temperature of 275 °C.

All spectra were acquired using an Orbitrap Fusion Tribrid mass spectrometer controlled by Xcalibur 2.0 software (Thermo Scientific) and operated in data-dependent acquisition mode using an SPS-MS3 workflow.

The raw data files were processed and quantified using Proteome Discoverer software v1.4 (Thermo Scientific) and searched against a database comprised of proteins predicted by the metagenome and metatranscriptome datasets, using SEQUEST [59]. Peptide precursor mass tolerance was set at 10 ppm, and MS/MS tolerance was set at 0.6 Da. Search criteria included oxidation of methionine, carbamidomethylation of cysteine and the addition of the TMT mass tag to peptide N-termini and lysine as fixed modifications. The reverse database search option was enabled, and all peptide data was filtered to satisfy false discovery rate (FDR) of 5%. Peptides were collapsed to protein groups which expressions are represented by their summed peptide median intensity. Expression values were log_10_ transformed and standardised by median centering and median absolute deviation (MAD) for scaling [60]. To identify significantly differentiated proteins between symptomatic and non-symptomatic samples, we performed a two-sample moderated *t* test. Raw peak intensity values of the experiment were deposited at the PRIDE repository [61].

## Additional files

Additional file 1: Table S1. Metagenomic gene annotation of sample AT2. MG-RAST gene predictions for sample AT2, based on the Swissprot database. MG-RAST ID 4702452.3. (XLSX 497 kb)

Additional file 2: Table S2. Metagenomic gene annotation of sample AT3. MG-RAST gene predictions for sample AT3, based on the Swissprot database. MG-RAST ID 4702456.3. (XLSX 470 kb)

Additional file 3: Table S3. Metagenomic gene annotation of sample AT4. MG-RAST gene predictions for sample AT4, based on the Swissprot database. MG-RAST ID 4702454.3. (XLSX 471 kb)

Additional file 4: Table S4. Metagenomic gene annotation of sample AT5. MG-RAST gene predictions for sample AT5, based on the Swissprot database. MG-RAST ID 4702457.3. (XLSX 240 kb)

Additional file 5: Table S5. Metagenomic gene annotation of sample AT8. MG-RAST gene predictions for sample AT8, based on the Swissprot database. MG-RAST ID 4702453.3. (XLSX 355 kb)

Additional file 6: Table S6. Metagenomic gene annotation of sample ROW1. MG-RAST gene predictions for sample ROW1, based on the Swissprot database. MG-RAST ID 4702451.3. (XLSX 515 kb)

Additional file 7: Table S7. Metagenomic gene annotation of sample ROW1–2. MG-RAST gene predictions for sample ROW1-2, based on the Swissprot database. MG-RAST ID 4702458.3. (XLSX 277 kb)

Additional file 8: Table S8. Metagenomic gene annotation of sample ROW2. MG-RAST gene predictions for sample ROW2, based on the Swissprot database. MG-RAST ID 4702455.3. (XLSX 564 kb)

Additional file 9: Table S9. Comparisons of metagenomic functional gene categories. Gene counts for MG-RAST SEED subsystem categories for all samples in this study. (XLSX 595 kb)

Additional file 10: Table S10. Common core metagenome of AOD symptomatic tissue. Genes identified in all symptomatic samples (AT5, AT8, ROW1, ROW1–2 and ROW2), and in no non-symptomatic samples. Based on MG-RAST annotation using Swissprot as reference database. (XLSX 105 kb)

Additional file 11: Table S11. Common core metagenome of non-symptomatic samples. Genes identified in all non-symptomatic samples (AT2, AT3 and AT4), and in no symptomatic samples. Based on MG-RAST annotation using Swissprot as reference database. (XLSX 64 kb)

Additional file 12: Table S12. Constructed bacterial genomes of this study. List of constructed genomes based on metagenomic read binning using the MetaBat software, their assigned genera and ID used in this study, and corresponding CheckM software analysis results. (XLSX 10 kb)

Additional file 13: Narrowed-down database containing all predicted and annotated genes of all organisms used in the specialised data analysis of this study. (FASTA 46859 kb)

Additional file 14: Table S13. Metatranscriptomic gene annotation of sample AT2. Identified unique genes in the metatranscriptome of sample AT2, using transcripts assembled using Trinity and annotating using the Trinotate pipeline. Swissprot was used as reference database. (XLSX 8417 kb)

Additional file 15: Table S14. Metatranscriptomic gene annotation of sample AT3. Identified unique genes in the metatranscriptome of sample AT3, using transcripts assembled using Trinity and annotating using the Trinotate pipeline. Swissprot was used as reference database. (XLSX 1326 kb)

Additional file 16: Table S15. Metatranscriptomic gene annotation of sample AT4. Identified unique genes in the metatranscriptome of sample AT4, using transcripts assembled using Trinity and annotating using the Trinotate pipeline. Swissprot was used as reference database. (XLSX 1219 kb)

Additional file 17: Table S16. Metatranscriptomic gene annotation of sample AT5. Identified unique genes in the metatranscriptome of sample AT5, using transcripts assembled using Trinity and annotating using the Trinotate pipeline. Swissprot was used as reference database. (XLSX 1134 kb)

Additional file 18: Table S17. Metatranscriptomic gene annotation of sample AT8. Identified unique genes in the metatranscriptome of sample AT8, using transcripts assembled using Trinity and annotating using the Trinotate pipeline. Swissprot was used as reference database. (XLSX 664 kb)

Additional file 19: Table S18. Metatranscriptomic gene annotation of sample ROW1. Identified unique genes in the metatranscriptome of sample ROW1, using transcripts assembled using Trinity and annotating using the Trinotate pipeline. Swissprot was used as reference database. (XLSX 1123 kb)

Additional file 20: Table S19. Metatranscriptomic gene annotation of sample ROW1–2. Identified unique genes in the metatranscriptome of sample ROW1–2, using transcripts assembled using Trinity and annotating using the Trinotate pipeline. Swissprot was used as reference database. (XLSX 1349 kb)

Additional file 21: Table S20. Common core metatranscriptome of AOD symptomatic tissue. Identified genes common to all symptomatic samples, but unidentified in non-symptomatic samples. Based on transcripts assembled using Trinity and annotated using Trinotate. Swissprot was used a reference database. (XLSX 1420 kb)

Additional file 22: Table S21. Common core metatranscriptome of non-symptomatic tissue. Identified genes common to all non-symptomatic samples, but unidentified in symptomatic samples. Based on transcripts assembled using Trinity and annotated using Trinotate. Swissprot was used a reference database. (XLSX 22 kb)

Additional file 23: Table S22. Comparative gene expression analysis of metatranscriptomes. List of genes identified as significantly altered in expression comparing the metatranscriptomes of symptomatic to non-symptomatic samples, using the voom function in R (limma package). Reference database for gene annotations was Swissprot. (XLSX 37 kb)

Additional file 24: Table S23. Geneset enrichment analysis results. Genesets identified as significantly differently expressed when comparing symptomatic to non-symptomatic samples, using the GSEA module of the GenePattern software (Broad Institute). (XLSX 1847 kb)

Additional file 25: Table S24. Metaproteome analysis of all samples on peptide level. Peptide level read output from Proteome Discoverer software (Thermo Fisher Scientific) version 2.1 for each protein, with a confidence FDR < 0.05. (XLSX 60 kb)

Additional file 26: Table S25. Constructed narrowed-down database. Dataset of annotated genes associated with *Quercus robur* and *Quercus petraea*, as found on the NCBI website. (XLSX 12886 kb)

Additional file 27: Table S26. Narrowed-down metatranscriptome read hits of sample AT2. Gene-read hits assigned using the database depicted in ‘Additional file 13’ and the Trinotate pipeline for sample AT2. (XLSX 7618 kb)

Additional file 28: Table S27. Narrowed-down metatranscriptome read hits of sample AT3. Gene-read hits assigned using the database depicted in ‘Additional file 13’ and the Trinotate pipeline for sample AT2. (XLSX 9021 kb)

Additional file 29: Table S28. Narrowed-down metatranscriptome read hits of sample AT4. Gene-read hits assigned using the database depicted in ‘Additional file 13’ and the Trinotate pipeline for sample AT2. (XLSX 2628 kb)

Additional file 30: Table S29. Narrowed-down metatranscriptome read hits of sample AT5. Gene-read hits assigned using the database depicted in ‘Additional file 13’ and the Trinotate pipeline for sample AT2. (XLSX 7176 kb)

Additional file 31: Table S30. Narrowed-down metatranscriptome read hits of sample AT8. Gene-read hits assigned using the database depicted in ‘Additional file 13’ and the Trinotate pipeline for sample AT8. (XLSX 16212 kb)

Additional file 32: Table S31. Narrowed-down metatranscriptome read hits of sample ROW1. Gene-read hits assigned using the database depicted in ‘Additional file 13’ and the Trinotate pipeline for sample AT2. (XLSX 11317 kb)

Additional file 33: Table S32. Narrowed-down metatranscriptome read hits of sample ROW1–2. Gene-read hits assigned using the database depicted in ‘Additional file 13’ and the Trinotate pipeline for sample AT2. (XLSX 65 kb)

Additional file 34: Figure S1. Analysis of metatranscriptomes using a curated database of abundant bacteria in the AOD lesion microbiome reveals that *Brenneria goodwinii* is the key pathogen, but assisted by others**.** A: *Brenneria goodwinii* (Bg in the figure) virulence-associated genes significantly upregulated in symptomatic samples compared to non-symptomatic samples. Genes were determined to be significantly different in expression by using the limma package in R (statistical cutoff at FDR < 0.05, and log_2_ cutoff at > 2). Circles in light grey going out from the centre indicate log_2_ fold changes, with the ratio numbers indicated to the side of the plots. Genes were separated into different colour coded categories. B: Oak tree defence-associated genes upregulated in symptomatic samples compared to non-symptomatic samples, based on our in-house annotated oak transcriptome database. (TIFF 946 kb)

Additional file 35: Table S33. Differentially expressed genes in AOD symptomatic tissue. List of significant genes resulting from comparative analysis of the non-symptomatic samples and symptomatic samples using the voom function of the R-package limma, based on an FDR value < 0.05 and log^2^-fold change of <− 2 and > 2. These results were generated on read hits against our narrowed-down database (Additional file 13). (XLSX 860 kb)

Additional file 36: Table S34. Metaproteome analysis of all samples on protein level using the narrowed-down database. Peptide level read output from Proteome Discoverer software (Thermo Fisher Scientific) version 2.1 for each protein, with a confidence FDR < 0.05. (XLSX 668 kb)

Additional file 37: Table S35. Comparative proteome results of genes of interest. List of all annotated proteins of interest using our narrowed-down database (Additional file 13). (XLSX 28 kb)

Additional file 38: Figure S2. Metaproteome analysis using a curated database of abundant bacteria in the AOD lesion microbiome confirms the dominance of *Brenneria goodwinii* in AOD. Using our narrowed-down database, we re-performed comparative metaproteomics of our non-symptomatic vs. symptomatic samples. Genes were determined to be significantly different in abundance using a student’s t-test. Gene names in all capital letters correspond to oak host genes, while belong to *B. goodwinii*. Categories of interest are depicted in the colour key. Genes with an abundance fold change of 15 were not detected in non-symptomatic tissue. Circles in light grey going out from the centre indicate the different log_2_ fold changes, with the numbers indicated to the side of the plots. (TIFF 435 kb)

Additional file 39: Table S36. List of all identified unique genetic features based on the Swissprot database in each sample, divided into data derived from Metagenome, Metatranscriptome and Metaproteome. (XLSX 868 kb)
