# Supplementary figures and images for: Integrated multi-omic analysis of host-microbiota interactions in acute oak decline
Source: Microbiome. 2018 Jan 30;6:21. doi: 10.1186/s40168-018-0408-5 (PMC5789699; doi:10.1186/s40168-018-0408-5)

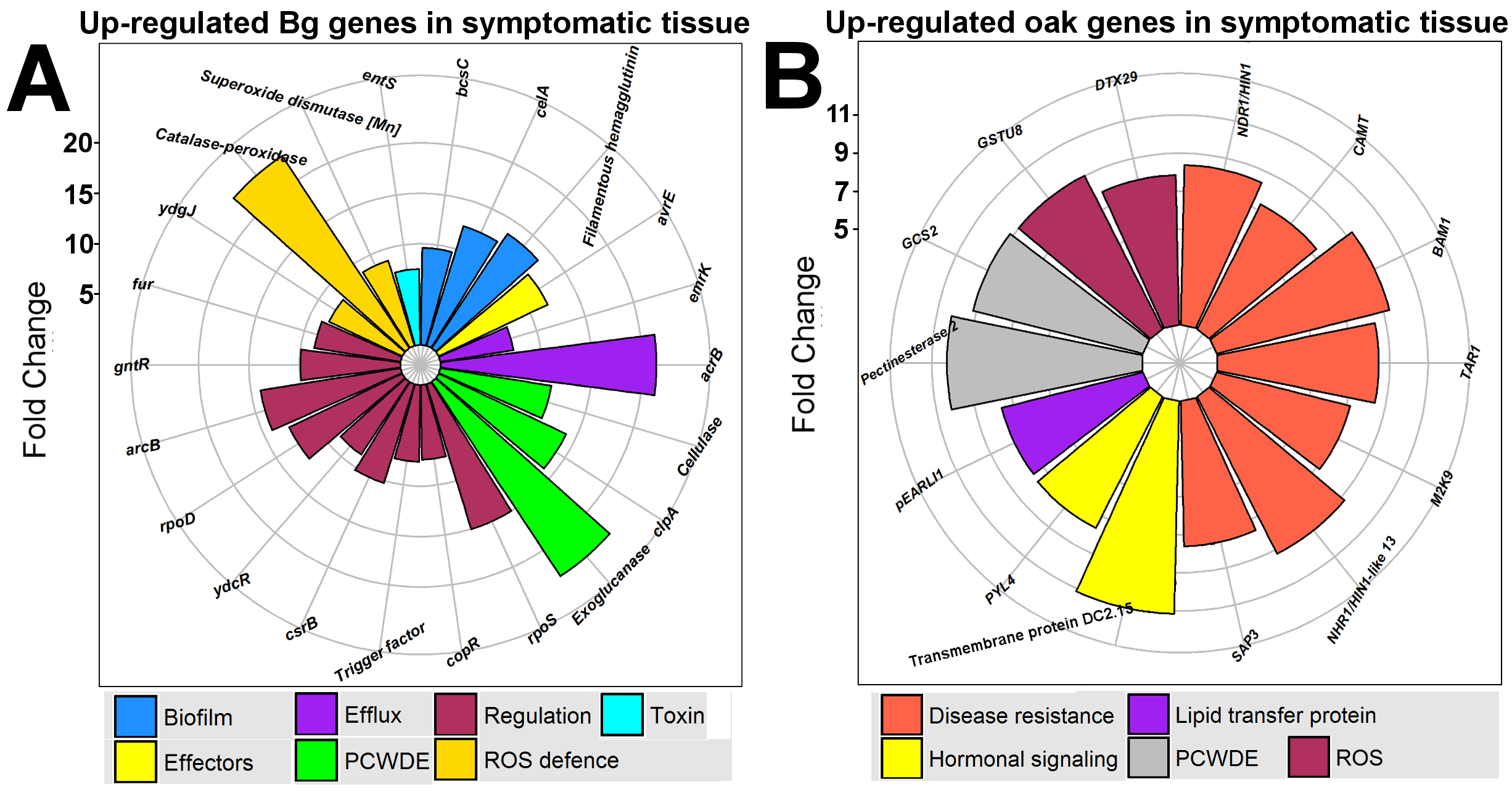

Supplement: Supplementary file 34 — Analysis of metatranscriptomes using a curated database of abundant bacteria in the AOD lesion microbiome reveals that Brenneria goodwinii is the key pathogen, but assisted by others. A: Brenneria goodwinii (Bg in the figure) virulence-associated genes significantly upregulated in symptomatic samples compared to non-symptomatic samples. Genes were determined to be significantly different in expression by using the limma package in R (statistical cutoff at FDR < 0.05, and log2 cutoff at > 2). Circles in light grey going out from the centre indicate log2 fold changes, with the ratio numbers indicated to the side of the plots. Genes were separated into different colour coded categories. B: Oak tree defence-associated genes upregulated in symptomatic samples compared to non-symptomatic samples, based on our in-house annotated oak transcriptome database. (TIFF 946 kb) [file 40168_2018_408_MOESM34_ESM.tif]

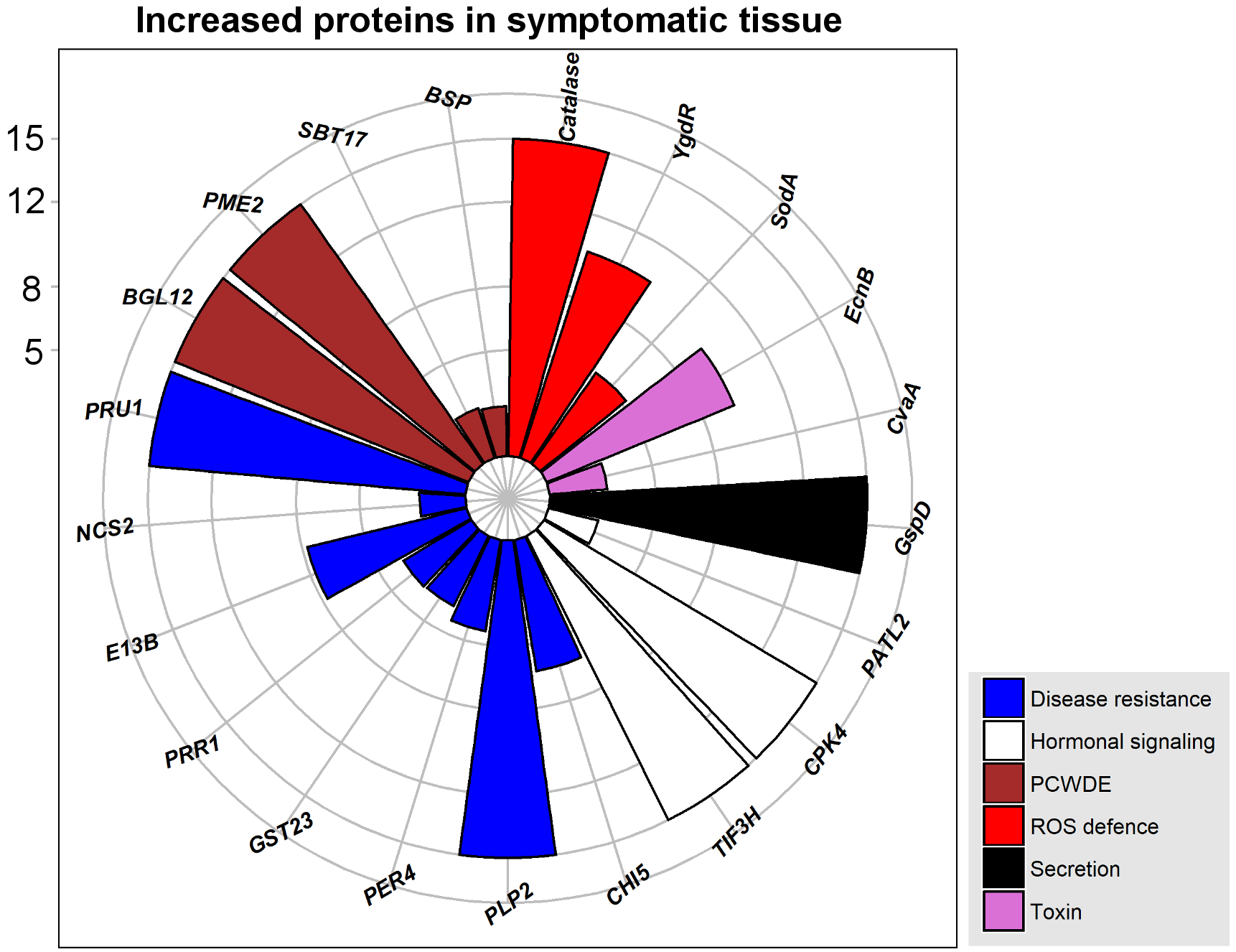

Supplement: Supplementary file 38 — Metaproteome analysis using a curated database of abundant bacteria in the AOD lesion microbiome confirms the dominance of Brenneria goodwinii in AOD. Using our narrowed-down database, we re-performed comparative metaproteomics of our non-symptomatic vs. symptomatic samples. Genes were determined to be significantly different in abundance using a student’s t-test. Gene names in all capital letters correspond to oak host genes, while belong to B. goodwinii. Categories of interest are depicted in the colour key. Genes with an abundance fold change of 15 were not detected in non-symptomatic tissue. Circles in light grey going out from the centre indicate the different log2 fold changes, with the numbers indicated to the side of the plots. (TIFF 435 kb) [file 40168_2018_408_MOESM38_ESM.tiff]
